# Client Factors Affect Provider Adherence to Clinical Guidelines during First Antenatal Care

**DOI:** 10.1371/journal.pone.0157542

**Published:** 2016-06-20

**Authors:** Mary Amoakoh-Coleman, Irene Akua Agyepong, Nicolaas P. A. Zuithoff, Gbenga A Kayode, Diederick E Grobbee, Kerstin Klipstein-Grobusch, Evelyn K Ansah

**Affiliations:** 1 Julius Global Health, Julius Center for Health Sciences and Primary Care, University Medical Centre Utrecht, The Netherlands; 2 School of Public Health, University of Ghana, Legon, Ghana; 3 Division of Epidemiology and Biostatistics, School of Public Health, Faculty of Health Sciences, University of the Witwatersrand, Johannesburg, South Africa; 4 Research and Development Division, Ghana Health Service, Accra, Ghana; Centers for Disease Control and Prevention, UNITED STATES

## Abstract

**Background:**

The first antenatal clinic (ANC) visit helps to distinguish pregnant women who require standard care, from those with specific problems and so require special attention. There are protocols to guide care providers to provide optimal care to women during ANC. Our objectives were to determine the level of provider adherence to first antenatal visit guidelines in the Safe Motherhood Protocol (SMP), and assess patient factors that determine complete provider adherence.

**Methods:**

This cross-sectional study is part of a cohort study that recruited women who delivered in eleven health facilities and who had utilized antenatal care services during their pregnancy in the Greater Accra region of Ghana. A record review of the first antenatal visit of participants was carried out to assess the level of adherence to the SMP, using a thirteen-point checklist. Information on their socio-demographic characteristics and previous pregnancy history was collected using a questionnaire. Percentages of adherence levels and baseline characteristics were estimated and cluster-adjusted odds ratios (OR) calculated to identify determinants.

**Results:**

A total of 948 women who had delivered in eleven public facilities were recruited with a mean age (SD) of 28.2 (5.4) years. Overall, complete adherence to guidelines pertained to only 48.1% of pregnant women. Providers were significantly more likely to completely adhere to guidelines when caring for multiparous women [OR = 5.43 (1.69–17.44), *p<0*.*01*] but less likely to do so when attending to women with history of previous pregnancy complications [OR = 0.50 (0.33–0.75), *p<0*.*01*].

**Conclusion:**

Complete provider adherence to first antenatal visit guidelines is low across different facility types in the Greater Accra region of Ghana and is determined by parity and history of previous pregnancy complication. Providers should be trained and supported to adhere to the guidelines during provision of care to all pregnant women.

## Introduction

Efforts by the World Health Organization (WHO) to facilitate attainment of the Millennium Development Goals (MDGs) 4 & 5, to reduce child and maternal mortality, have included provision of evidence-based guidelines to ultimately improve quality of care [1–3] and these have been adopted by member countries for implementation.

### Antenatal Care

Antenatal care is a preventive and promotive health service provided to pregnant women, with the goal of preventing, detecting and alleviating the health problems that affect mothers and babies during pregnancy[4]. The full life-saving potential that antenatal care promises for women and babies is realized with a minimum of four visits during the pregnancy, within the context of focused antenatal care [4;5]. Some of the services to be provided include but are not limited to taking the history of the woman; examination of the woman’s general and obstetric wellbeing; screening for specific diseases and other conditions; preventive measures and therapies, treatment for existing conditions, as well as health education and counseling. These measures are applicable even at the lowest level of care [4]. For the antenatal care interventions to work effectively, it is important that the first antenatal clinic (ANC) visit takes place as early as possible in pregnancy, preferably in the first trimester. Although most maternal deaths occur during labour, delivery, or during the first 24 hours after delivery [6], those that are likely to have problems at birth can be identified during the antenatal period[7]. The first ANC visit is to identify and distinguish pregnant women who require standard care, from those with specific risk factors and so require special attention [4]. In Ghana, the guidelines for the first antenatal visit as per the Safe Motherhood Protocol (SMP) include history taking that covers age, parity, gestational age at booking, and history of last pregnancy where applicable. Examination, comprised of measuring of the weight and blood pressure of the pregnant woman as well as abdominal examination are also required. Urine and hemoglobin tests are to be carried out and finally, administration of iron supplements, tetanus injections and sulfadoxine pyrimethamine for intermittent preventive therapy for malaria (IPTp) [8]. These requirements are in agreement with what is prescribed in the national Standard Treatment Guidelines (STG). The maternal health record book [9] which bears the records of the woman’s ANC visits is also designed to capture all the requirements in the guidelines.

### Adherence to Clinical Guidelines

The quality of clinical decision making by frontline providers of maternal and new-born care is among the factors retarding Ghana’s progress towards the attainment of the Millennium Development Goals 4&5 [10;11]. The health professionals’ attitudes towards risk can lead to significant variations in the way that decisions regarding patient care are made [12]. Clinical guidelines are set to ensure and assure uniformity as well as the quality of provision of care [13]. Utilization of guidelines is admittedly complex, and influenced by many factors [14]. Adherence has been defined as “conformity to, fulfilling or following official, recognized or institutional requirements, guidelines, recommendations, protocols, pathways or standards” [15]. In clinical practice this translates into the extent to which care-giving practice conforms to recognized and evidence-based standards [16–19]. Adherence studies have shown varying levels of adherence to clinical practice guidelines, with data pointing to relatively lower levels of adherence [20;21]. Many reasons have been assigned for non-adherence to guidelines [22;23]. Some of these reasons include but are not limited to, physicians’ unawareness of recommendations, their disagreement with the guidelines and their perceptions that the guidelines are not easily applicable in daily practice, amongst others [1;24;25]. There is currently no information in Ghana on how adherent providers are to available antenatal care guidelines like the SMP. Direct patient factors influencing provider adherence have also not been extensively studied. In this paper, our aim is to document the level of provider adherence, as well as assess patient factors that determine the level of provider adherence during the first ANC visit at different levels of care [1].

## Materials and Methods

### Study design and setting

A cross-sectional analysis of the baseline data of a prospective cohort study on adherence to protocol and how it affects pregnancy outcomes, was conducted in eleven health facilities in the Greater Accra Region (GAR) of Ghana.

The Greater Accra Region (GAR) has a total of twenty health administrative districts and sub-metropolises and is served by both public and private facilities. The public sector comprises of one teaching hospital, a regional hospital and nine district and sub-metropolitan hospitals. There are ten polyclinics, thirty-one health centers, some community clinics and three Community Health Planning and Services (CHPS) compounds. The polyclinics are primary health care facilities like the health centers, usually sited in urban and populous areas and provide services for different conditions under different units. They are run by non-specialist medical officers and medical or physician assistants, together with midwives, nurses and other paramedical staffs. Cases are referred from here to the district hospitals. The CHPS compounds operate at the community level, with midwives or community health nurses, either through home visits or clients going to the compounds. They provide basic close-to-client primary care services like health education, treatment of minor ailments and injuries, antenatal services, normal deliveries, and postnatal services. There are many private hospitals operating in the region and some of these serve as district hospitals in the districts without a public hospital. All these health facilities offer antenatal and delivery services, though the smaller facilities refer complications during pregnancy and delivery to the bigger health facilities. The National Health Insurance Scheme (NHIS) is operational in all the public as well as in most of the private facilities.

### Sample size

The sample size for the cohort study which was computed using Open Epi calculator [26], based on a prevalence of pregnancy complications of 6% [27]. We assumed that the complication rate will be twice as high amongst the exposed group (incomplete adherence). To detect a two-sided significance difference at 95% confidence interval, at a power of 80%, and a one-to-one ratio of exposure to non-exposure, a sample of 372 women was required per exposure group. The total sample size therefore required for both arms was 744.

### Selection of districts and facilities

We desired to have participants randomly selected from the different levels of care (variable “type of facility”) across the Greater Accra region. In addition to the regional hospital, ten health facilities (hospitals, polyclinics and a health center) were randomly selected for the study. Initially, five out of the eight districts or sub-metropolises with hospitals were selected by balloting with replacement for inclusion of their district or sub-metropolitan hospitals. Another five of the remaining twelve districts (which have no public hospitals) were selected by the same procedure for inclusion of a primary level facility offering both antenatal and delivery services. Thus in all 11 facilities (clusters) were used.

### Variables

The outcome variable studied was provider adherence, defined as complete or incomplete (moderate or poor).

The determinants were type of facility, client’s socio-demographics (age, educational level, marital status and employment status), and client’s prenatal factors (parity, trimester at first ANC visit, previous pregnancy history and number of times ANC was attended during pregnancy).

#### Measuring adherence

Several studies have measured adherence using a scoring system, based on available guidelines or protocol requirements [22;28–30]. A thirteen-point checklist was used to score provider adherence to first antenatal visit guidelines. The questions on the checklist were based on the requirements for first ANC visit as per the SMP for Ghana, which is also consistent with the national treatment guidelines for first ANC visit. Two of the variables on the checklist, herein referred to as “optional” variables, may not be due at the first ANC visit, depending on the woman’s gestational age and therefore do not influence the adherence categorization. These are the “last pregnancy history if applicable” and “Intermittent Presumptive Treatment in pregnancy (IPTp) given if woman is due”. IPTp is indicated for women in the second and third trimesters only. The optional variables were included to help us assess their influence on quality of antenatal care. The remaining eleven variables are required for all clients irrespective of the gestational age (“age”, “parity”, “gestational age at booking”, “medical, surgical or family history”, “weight”, “blood pressure”, “abdomen examination”, “hemoglobin test”, “urine test”, “iron supplement”, “tetanus injection”, and are herein referred to as “mandatory” variables). Every record reviewed was assessed to see how many of the thirteen variables were actually adhered to by the provider at the first ANC visit. Each variable adhered to, scored a point of 1 while non-adherence scored 0. Scoring for adherence was based on only the mandatory variables. A total score of 11 to all mandatory variables was classified as complete adherence to guidelines. Non adherence to any of the mandatory variables was classified as incomplete adherence. Incomplete adherence was re-categorized into *moderate* (score of 9–10) and *poor* (score less than 9) adherence during data analysis. S1 Table describes the variables on the checklist and the scoring criteria.

### Recruitment of women

Participants were recruited at delivery and once they met the inclusion criteria, informed consent was obtained. The inclusion criteria included the following: participant 18 years or older; participant has had at least one ANC visit in a health facility during the current pregnancy; participant had first ANC visit at gestational age less than or equal to 5 months and participant had first ANC visit at the facility of delivery or in one of the sampled facilities for this study.

From each of the eleven [11] facilities sampled for the study, we recruited a minimum of 68 women, 34 exposed to non-adherence (incomplete adherence) and 34 unexposed (complete adherence). We needed to ensure that the minimum number of clients with the required exposure status was satisfied. The initial assumption was that the exposed (non-adherence) would be less than the unexposed. That was the basis for the decision to recruit till a minimum of 34 exposed per facility were recruited. However, during the conduct of the study we noticed that the exposed were rather more and so we had to recruit till a minimum of 34 unexposed were recruited. The minimum of 34 participants per exposure status was therefore satisfied. This also enabled the estimation of the prevalence of adherence to the guidelines at baseline among the study participants.

### Data collection processes and tools

Every woman delivering at the facility on any day and who met the inclusion criteria, and provided consent for participation, was enrolled into the study.

At recruitment, a record review of their first ANC visit from the maternal health record book was carried out using a checklist. Data on their socio-demographic characteristics, potential confounders as well as the 13 variables on guideline requirements were collected. Records of subsequent ANC visits were also reviewed for any complications developed and identified during the pregnancy. Finally, data on the delivery outcomes was collected from both the maternal health record book and delivery register and notes. Participants’ telephone numbers were linked to their study identification numbers (IDs) for follow up.

The women and their neonates were followed up till 6 weeks postpartum to complete data collection on outcomes. Follow-up was at the health facility during postnatal care and also by phone. Those who could not be reached by any of these means were treated as lost to follow up, but this loss to follow-up was expected to be minimal and was taken into consideration during the sample size calculation. A facility audit was conducted to assess facility factors such as the availability of personnel, services, infrastructure, logistics and supplies that are needed to support adherence to the guidelines at facility level.

The assumption was made that any information on history, examination, laboratory examination and treatment available is what was recorded in the maternal health record book. Information on any service not recorded, was deemed not to have been delivered. *(WHO recommends that at the first visit*, *all such information should be recorded in the client’s record book)*

### Data Analysis

Descriptive analysis of participants’ socio-demographic information was carried out. Adherence to guidelines was computed by calculating the proportion of women whose first ANC visits met the criteria for complete adherence. Incomplete adherence (exposure) was re-categorized into moderate and poor adherence status to assess whether different extents of non-adherence affect the association with the determinants. The association between determinants was analyzed with a generalized linear model for dichotomous outcomes. An exchangeable residual (i.e. the generalized estimation equation type) covariance matrix was incorporated to correct for a potential cluster effect, which may arise due to our sampling strategy, which involved 11 facilities (clusters) [31]. Associations were estimated with odds ratios with their 95% confidence intervals (CI), significance tests were based on Wald chi-square tests. P-values < 0.05 were considered significant. We tested a model with all potential confounders, except “number of times ANC was attended” since this cannot be known at the time of the first ANC visit and so cannot be a determinant of provider adherence at that time. Data analysis was carried out using IBM SPSS Statistics for Windows, Version 20.0. Armonk, NY: IBM Corp.

### Ethical considerations

The protocol, which included a written consent document, was submitted to the Ghana Health Service (GHS) Ethical Review Committee (ERC) and approval obtained under study ID No. GHS-ERC 12/07/2013 before the study was conducted. Approval from the district/ sub-metropolitan heads as well as the regional health directorate was also obtained. Written informed consent was obtained from each participant (pregnant women, heads of facilities and unit heads)' using the approved consent document.

## Results

Overall, nine hundred and forty-six women who had delivered in eleven public facilities were recruited into the study from December 2013 to April 2014. Fifty-six percent (531) of them were seen to at six district hospitals, while 36.0% (341) were from four polyclinics and 7.9% (74) from a health center.

### Participants’ characteristics: Demographic and socio-economic factors

The mean age (SD) of the women in the study was 28.2 (5.4) years. About eighty-five percent of them were within the 20–35 years age group. Half of participants (430) had secondary education, with 11.6% (109) and 19.9% (187) having no education and primary education respectively. Most of the women 644 (69.0%) first attended ANC during the second trimester and 78.2% (741) attended at least four times during the pregnancy. Married women comprised 74.2% (689) of all participants and most of the participants 797 (85.0%) were employed. Only 7.6% (72) of the women had a previous pregnancy complication (Table 1).

**Table 1:** Baseline characteristics of study participants and association of these characteristics with provider adherence levels to first ANC guidelines.

Variable	Variable category	Frequency (%) N = 946	% Poor adherence N = 130	% Moderate adherence N = 361	% Complete adherence N = 455	Cluster-adjusted p-value
Facility type						<0.01
	Hospital	531 (56.1)	14.3	38.0	47.6	
	Polyclinic	341(36.0)	7.9	40.2	51.9	
	Health center	74 (7.9	36.5	29.7	33.8	
Age category						0.11
	< 20 years	54 (5.7)	20.4	53.7	25.9	
	20–35 years	805 (85.3)	13.9	36.9	49.2	
	>35 years	85 (9.0)	7.1	41.2	51.8	
Parity						0.01
	0	264 (27.9)	23.5	53.8	22.7	
	1–2	485 (51.3)	8.0	32.8	59.3	
	3–4	164 (17.3)	12.8	31.1	56.1	
	>4	33 (3.5)	24.2	27.3	48.5	
Education						0.91
	None	109 (11.6)	11.0	36.7	52.3	
	Primary	187 (19.9)	14.4	34.2	51.3	
	Secondary	430 (45.7)	14.4	37.4	49.1	
	Tertiary	166 (17.7)	12.7	45.2	42.2	
	Other	48 (5.1)	8.3	39.6	52.1	
Trimester of 1^st^ ANC						0.03
	First	289 (31.0)	12.5	45.3	42.2	
	Second	644 (69.0)	13.8	34.8	51.4	
No. of times ANC attended						<0.01
	1	46 (4.9)	45.7	34.8	19.6	
	2–3	138 (14.6)	16.8	32.1	51.1	
	> = 4	741 (78.2)	11.2	39.3	49.5	
Marital status						0.01
	Single	102 (11.0)	13.7	51.0	35.3	
	Married	689 (74.2)	11.2	37.4	51.4	
	Formerly married	12 (1.3)	41.7	16.7	41.7	
	Living together	125 (13.5)	22.4	34.4	43.2	
Employment						0.02
	No	141 (15.0)	12.8	53.2	34.0	
	Yes	797 (85.0)	13.7	35.8	50.6	
Previous pregnancy complication						0.33
	No complication	872 (92.4)	14.2	37.4	48.4	
	Complication	72 (7.6)	8.3	48.6	43.1	
Mean age (SD)		28.2 (5.4)	26.7 (5.3)	27.4 (5.7)	29.1 (4.9)	<0.01

### Adherence to first ANC guidelines

At their first ANC visit, all 946 participants had their ages checked, 99.3% (939) had a urine test done, 99.0% (937) had their weight checked, 98.5% (932) had a hemoglobin test done, and 98.3% (930) had their blood pressure checked. A total of 916 (96.8%) participants had iron tablets prescribed for them while 86.3% (816) had their parity checked. Only 72.4% (685) had their abdomen examined while the dose of sulphadoxine pyrimethamine for prevention of malaria in pregnancy was administered to 581 (61.4%) participants.

The mean total adherence score (95% CI) out of a maximum of 13 was 11.7 (11.7–11.8). Mean percentage adherence per variable on the checklist was 90.3% (83.1–97.4%). Overall, complete adherence to guidelines pertained to only 48.1% of participants during their first ANC visit. Care for 38.2% (361) of women reflected moderate adherence to guidelines by providers while for 13.7% (130) of women there was poor adherence. Complete adherence to guidelines was higher amongst women seen at the polyclinics (51.9%) than those seen in hospitals (47.6%) and the health center (33.8%).

Adherence to the mandatory variable ‘abdominal examination’ was positively correlated with ‘gestational trimester at the first visit’ *(p<0*.*01)*, with 77.8% (501/644) whose first ANC visit was during the second trimester being examined compared to 59.9% (173/289) of women who first came during the first trimester. Adherence to the optional variable ‘IPTp given if woman is due’ was positively correlated with ‘gestational trimester at the first visit’ *(p<0*.*001)*, with 70.0% (451/644) of second trimester registrants given IPTp compared to 43.6% (126/289) first trimester registrants. Details of adherence scores are presented in Table 2.

**Table 2:** Summary of proportion of provider adherence to first ANC guidelines for 946 study participants.

Variable	Variable Category	Frequency (%) Yes / Checked
Checklist variable	Age	946 (100.0)
	Parity	816 (86.3)
	Gestational age at registration	862 (91.1)
	Last pregnancy history taken if applicable	807 (85.3)
	Medical history taken	930 (98.3)
	Weight recorded	937 (99.0)
	Blood pressure measured	930 (98.3)
	Abdomen examined	685 (72.4)
	Hemoglobin test done	932 (98.5)
	Urine test done	939 (99.3)
	Iron tablet given	916 (96.8)
	Tetanus injection given or status recorded	822 (86.9)
	IPTp given if due	581 (61.4)
Total adherence score		
	6	1 (0.1)
	7	4 (0.4)
	8	8 (0.8)
	9	20 (2.1)
	10	97 (10.3)
	11	273 (28. 8)
	12	212 (22.4)
	13	331 (35.0)
Adherence status		
	Complete	455 (48.1)
	Incomplete (moderate)	361 (38.2)
	Incomplete (poor)	130 (13.7)
Mean total adherence score (95% CI)		11.73 (11.65–11.81)
Mean % adherence per variable on checklist (95% CI)		90.27 (83.07–97.47)

### Baseline comparison of participants and adherence level groups

The mean age (SD) of the women whose care reflected complete adherence is 29.1 (4.9) years compared to 27.4 (5.7) and 26.7 (5.3) years respectively for those whose care reflected moderate and poor adherence levels respectively. Complete adherence levels for the different parity groups were significantly different (*p = 0*.*01)*. Differences in percentage complete adherence amongst the different educational levels were not significant (*p = 0*.*91*). Care for 331 out of 644 (51.6%) women attending their first ANC during the second trimester of pregnancy, reflected complete adherence compared to (122/289) 42.2% of those attending in first trimester. Complete adherence was reflected in the care of 70/138 (50.7%) women who attended ANC 2–3 times during pregnancy which was higher compared to level of adherence of the other groups, at *p < 0*.*01*. There was no significant difference between the levels of complete adherence between the care for women with and without previous pregnancy complication, both of which were below 50.0% (*p = 0*.*33*). Table 1 gives details of the comparability of the women in the three adherence level groups.

### Patient determinants of adequate provider adherence

In unadjusted univariate analysis, providers were more likely to completely adhere to first ANC guidelines when caring for multiparous women [OR = 4.68 (1.42–15.43), p = 0.01], employed women [OR = 2.1 (1.2–3.7) *p = 0*.*02*], married women [OR = 2.09 (1.18–3.71), p = 0.01] and women attending first ANC during the second trimester [1.4 (1.0–1.9) *p = 0*.*06*] (Table 3).

**Table 3:** Patient determinants of complete provider adherence to first ANC guidelines (Crude and adjusted odds ratios with p-values).

Variable	Variable category	OR (95%CI)	p-value	AOR* (95% CI)	p-value
Facility type					
	Hospital	1.00		1.00	
	Polyclinic	1.16 (0.74–1.09)	0.52	1.18 (0.56–2.49)	0.67
	Health center	0.55 (0.47–0.62)	<0.01	0.54 (0.40–0.73)	<0.01
Age					
	<20 years	1.00		1.00	
	20–35 years	2.90 (1.45–5.79)	<0.01	1.31 (0.59–2.90)	0.55
	>35 years	3.11 (1.11–8.71)	0.03	0.85 (0.43–1.70)	0.64
Trimester first ANC					
	First	1.00		1.00	
	Second	1.37 (0.99–1.90)	0.06	1.16 (0.72–1.87)	0.54
Education					
	None	1.00		1.00	
	Primary	0.99 (0.63–1.59)	0.99	0.92 (0.60–1.42)	0.70
	Secondary	0.87 (0.68–1.11)	0.26	1.02 (0.72–1.44)	0.93
	Tertiary	0.72 (0.50–1.04)	0.08	0.86 (0.56–1.33)	0.43
	Other	1.04 (0.55–1.34)	0.91	0.24 (0.69–2.24)	0.47
Parity					
	0	1.00		1.00	
	1–2	5.25 (1.54–17.91)	0.01	5.23 (1.59–17.26)	0,01
	3–4	4.68 (1.42–15.43)	0.01	5.43 (1.69–17.44)	<0.01
	>4	4.08 (1.28–12.97	0.02	4.77 (1.55–14.69)	0.01
Marital status					
	Single	1.00		1.00	
	Married	2.09 (1.18–3.71)	0.01	1.06 (0.59–1.88)	0.82
	Formerly married	1.89 (0.74–4.79)	0.19	1.06 (0.59–1.88)	0.13
	Living together	1.55 (0.74–3.21)	0.24	0.92 (0.52–1.61)	0.75
Employment					
	No	1.00		1.00	
	Yes	2.06 (1.15–3.68)	0.02	1.43 (0.92–2.24)	0.11
Previous history of pregnancy complication					
	No	1.00		1.00	
	Yes	0.83 (0.57–1.21)	0.33	0.50 (0.33–0.75)	<0.01

In a multivariable analysis that adjusted for client factors, providers were significantly more likely to completely adhere to guidelines when caring for multiparous women [OR = 5.43 (1.69–17.44), *p<0*.*01*] but less likely to do so when attending to women with previous pregnancy complications [OR = 0.50 (0.33–0.75), *p<0*.*01*] (Table 3).

## Discussion

### Main findings

We found complete adherence to first ANC guidelines level of 48.1% amongst our study participants. This is relatively low compared to complete adherence level of 54.5% found in a meta-analysis of adherence to practice guidelines for various medical and surgical conditions, as well as 55.9% at baseline of a cohort study in the USA [32;33]. It is however better than the estimate found in another study which reported 30.5% full or complete adherence level [34]. Another study found as high as 68% full adherence but this was in a prospective study and the authors admit possibility of Hawthorne effect[35]. We believe this level of complete adherence needs to be improved upon and education of providers about the guidelines and how they help improve service delivery outcomes will be essential.

The mandatory variable that was least adhered to was abdominal examination and we believe it is explained by its positive association with gestational trimester at the first visit. This supports the thinking of some providers who work in the study setting that when the gestational age is small, there is nothing significant to be palpated on abdominal examination. However this could be a missed opportunity since abdominal examination can corroborate the accuracy of the last menstrual period (LMP) data provided, and is also useful in assessing fetal viability towards the end of the first trimester. This may indicate the need for better training of or reminding providers in assessing gestational age by fundal height measurements and use of fetoscope. It must be noted that all women in our study had their first antenatal visit either during the first trimester or early second trimester as per the inclusion criteria.

The observed correlation between gestational trimester at first ANC visit and whether the woman received presumptive treatment for malaria may be due to the fact that sulphadoxine-pyrimethamine is prescribed only after the woman feels quickening or after 16 weeks gestation during the second trimester. It is thus surprising that about 40% of the women who first attended ANC during the first trimester were prescribed the medication. This could have serious consequences for the survival of the fetus as a result of congenital malformations in early life, due to the anti-folate nature of sulphadoxine pyrimethamine [36].

We did not evaluate the type of provider in relation to adherence levels. Whereas a previous study [34], has shown that care given by specialists usually conforms to practice guidelines, other studies have shown that providers at lower level facilities are more likely to follow guidelines[37]. In our study setting adherence levels were observed to be better in polyclinics compared to health centers and hospitals, although generally polyclinics tend not to have specialists present. This suggests that other factors, beyond the training of the provider, play a role in the level of the provider adherence. These factors could be further explored.

Anemia in pregnancy is common in developing countries, making routine iron supplementation necessary for every pregnant woman in these areas [38] The current study demonstrates excellent adherence to testing for hemoglobin at first visit (98.5%) and prescription of iron tablets (98%).

Patient factors have been found to be associated with provider adherence to guidelines [39;40]. We observed that provider complete adherence to the guidelines is determined by some patient factors like parity of the pregnant woman and history of previous pregnancy complication. Parity is known to be related to pregnancy outcomes, with multi-parity endangering outcomes [41;42]. It is therefore perhaps useful that multi-parity increases the likelihood of provider complete adherence to guidelines. Compromising quality of care by provider non-adherence to guidelines could result in dire consequences for both mother and neonate. It however seems counter-intuitive that in caring for women with positive history of previous pregnancy complication, providers tend to incompletely adhere to the first ANC guidelines. A similar finding was observed when providers were reported to be non-adherent to guidelines in caring for patients with severe form of disease [39]. Perhaps when there is such signal to danger, providers rather pay attention to the guideline items that relate to the woman’s specific problem, although it is unclear how this applies to the first antenatal visit. Guideline use seeks to ensure quality of care patients receive, with implications on care outcomes. It is therefore important that all women receive appropriate care as prescribed by the guidelines, irrespective of their status.

In general, complete adherence levels were observed to be low and it is therefore important that providers are engaged in a process to ascertain possible reasons for this finding and how best to improve it. Also, most women report late for first antenatal care and therefore public education and community based activities should be implemented to encourage women to seek ANC earlier.

### Strengths and limitations

This study retrospectively analyzed baseline data of a cohort of women so it is not possible that provider practice changed related to the study. The results reflect everyday provider practice and we believe this is strength of the study. As only women attending ANC and delivering in public facilities were included in the study, generalizability of the results to private practice is limited. We were also not able to gather data that explain other factors, especially from the provider’s perspective that might have influenced adherence to guidelines. We therefore recommend that these factors be explored in future qualitative studies.

## Conclusion

Complete provider adherence to first antenatal visit guidelines is low across different facility types in the Greater Accra region of Ghana and is determined by maternal parity and history of previous pregnancy complication. Since adherence to the guidelines at first visit has major implications for the quality of care of the pregnant woman providers should be trained to adhere to the guidelines during provision of care to all pregnant women.

## Supporting Information

S1 Table Table showing variables on adherence checklist for first ANC guidelines and scoring criteria. (DOCX)
