# Supplementary material for: Client Factors Affect Provider Adherence to Clinical Guidelines during First Antenatal Care
Source: PLoS One. 2016 Jun 20;11(6):e0157542. doi: 10.1371/journal.pone.0157542 (PMC4913935; doi:10.1371/journal.pone.0157542)
Supplement: S1 Table — (DOCX) [file pone.0157542.s001.docx]

**Supplement 1: Table showing variables on adherence checklist for first ANC guidelines and scoring criteria**

| Variable checked  **** Optional** variables  Others are **Mandatory** | Yes (1) | No (0) |
| --- | --- | --- |
| 1. Age recorded |  |  |
| 1. Parity recorded |  |  |
| 1. Gestational age at booking recorded |  |  |
| 1. Last pregnancy history if applicable recorded****** |  |  |
| 1. Medical, surgical or family history recorded |  |  |
| 1. Weight recorded |  |  |
| 1. Blood pressure recorded |  |  |
| 1. Abdomen examined |  |  |
| 1. Hemoglobin test done |  |  |
| 1. Urine test done |  |  |
| 1. Iron supplement given |  |  |
| 1. Tetanus injection given or status recorded |  |  |
| 1. Intermittent preventive treatment of malaria (IPTp) given if woman is due ** |  |  |
| **Adherence Score**  Total for Mandatory =  Total for Optional =  Overall Total = |  |  |
| **Adherence status (using mandatory variables)**  Total score = 11 …………………………………………………………... **Complete adherence**  Total score = 9-10………………………………………………………….. **Moderate adherence Incomplete**  Total score = 0-8 …………………………………………………………….. **Poor adherence adherence** | | |
